# Effects of Laser Forming on the Mechanical Properties and Microstructure of DP980 Steel

**DOI:** 10.3390/ma15217581

**Published:** 2022-10-28

**Authors:** Wenbin Dong, Le Bao, Wenqi Li, Kyoosik Shin, Changsoo Han

**Affiliations:** 1 Department of Mechatronics Engineering, Hanyang University, Ansan 15588, Gyeonggi-do, Korea; 2 Department of Mechanical Engineering, Anhui Science and Technology University, Chuzhou 233100, China

**Keywords:** DP980 steel, strength, microhardness, elongation, microstructure, laser forming

## Abstract

Due to its high strength and good plasticity, dual-phase (DP) steel is widely used for manufacturing the structural and reinforcement components of automobiles. Therefore, it is urgent to investigate the mechanical properties and microstructure variation in DP steel after deformation, especially those subjected to hot-forming processes. In this study, the mechanical properties and microstructure of laser-formed DP980 steel plates under different laser parameters were investigated by means of monotonic tensile tests, microhardness tests, and metallographic tests. The results showed that both yield strength and tensile strength increased with increasing laser line energy in the range of 5~19 J/mm due to the increasing volume content of martensite laths. Elongation was slightly improved after the laser-forming process due to the existence of residual austenite. The average microhardness of the heat-affected zone also increased with an increase in laser line energy and reached a maximum of 412.8 HV_0.2_—an improvement of 23.5% compared to that of the parent material.

## 1. Introduction

Dual-phase (DP) steel is a kind of advanced high-strength steel (AHSS) that is obtained by some specific heat treatments of low-carbon steel. The microstructure of DP steel consists mainly of ferrite and martensite, which give the material good plasticity and high strength [1,2]. Currently, DP steel is widely used for manufacturing automobile components to achieve lightweight vehicles. It was reported that DP steel accounted for 74% of the total weight of an auto body in the ULSAB-AVC (UltraLight Steel Auto Body-Advanced Vehicle Concepts) program of the USA [3]. As a typical representative of DP steel, DP980 steel is usually employed for manufacturing the structural and reinforcement components of an automobile, such as the girder, suspension system, seat rail, and bumper [4,5]. These components play important roles not only in reducing weight but also automobile safety. Therefore, it is of great significance to understand changes in the mechanical properties and microstructure of DP980 steel after deformation. A number of researchers have studied the static and dynamic mechanical properties of DP980 steel and established its constitutive model. Tian et al. [1], Xu et al. [6] and Zhang et al. [7] confirmed that the yield strength, tensile strength and elongation of the DP980 steel are not sensitive to the strain rate change in the lower strain rate range of 10^−^^3^ s^−^^1^ to 10^−^^1^ s^−^^1^, but they are significantly enhanced with an increase in the strain rate in the higher range of 10^0^ s^−^^1^ to 10^3^ s^−^^1^. Ren et al. [8] compared the strength variation characteristics among DP590, DP780 and DP980. They found that the strain rate sensitivity of DP980 is the lowest and DP590 the highest. However, due to the high strength of DP980 steel, the springback phenomenon is difficult to avoid during plastic deformation. Most previous studies on the forming of DP980 steel focused on springback prediction and formability improvement. Liu et al. [9] predicted the springback of DP980 steel in cold roll forming, indicating that the predictions were more accurate when using a nonlinear elastic modulus model rather than a constant one. Cao et al. [10] studied the forming and springback characteristics of a closed anti-collision beam made of DP980 steel and predicted the springback of a beam with a complex cross-section. Lee et al. [11] predicted the springback of a DP980 steel sheet in forming a seat-side frame with die compensation. Choi et al. [12] studied the tensile properties and fracture-forming limits of DP980 steel and then proposed a feasible approach of combining deep drawing and electromagnetic sharp edge forming to improve the formability of DP980 steel sheets. The authors and their research group [13,14,15] proposed a laser-forming process to significantly reduce springback and improve the formability of DP980 steel sheets. Laser forming is a novel technology of bending metal plates by nonuniform thermal stress coming from laser-heating cycles, with the advantages of no rigid tools or external forces. The springback of the laser-formed components is also negligible [16,17,18,19]. However, during the laser-forming process, changes in the mechanical properties and microstructure of the material occur with the huge temperature variation, which must be taken into account in the manufacturing of key components.

In this study, the mechanical properties of a laser-formed DP980 steel plate under different laser parameters were investigated by means of monotonic tensile tests and microhardness tests. Metallographic tests were also performed to understand the microstructural variation in the material.

## 2. Materials and Methods

### 2.1. Laser-Forming Experiment

**Figure 1:**
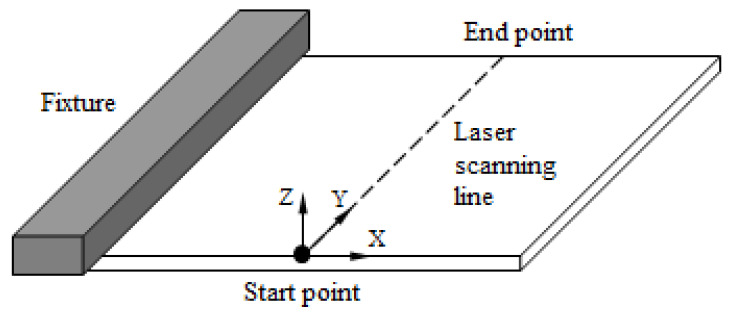
Schematic view of the laser-forming experiment setup.

The specimens used in the present experiment were square DP980 high-strength steel plates with dimensions of 80 mm×80 mm×1.8 mm. The specimens were clamped at one side, and a laser beam scanned them along the center line of the top surface, as shown in Figure 1. A YLR-150/750-QCW-AC laser system manufactured by IPG company in Burbach, Germany was employed for the experiment, with an output power range of 30~280 W and a laser wavelength of 1070 nm. The diameter of the laser spot was 2.5 mm. Nine combinations of laser parameters were selected to investigate their effects on the microstructure and mechanical properties, which are listed in Table 1. Line energy refers to the ratio of the laser energy absorption capacity of the steel plate to the laser-scanning velocity [20], which is calculated by Equation (1).

(1) $$E=\eta \frac{P}{v}$$

where, E, η, P and v denote the line energy, absorptivity, laser power and laser-scanning velocity, respectively. According to the experiment, η takes the value of 0.75.

### 2.2. Monotonic Tensile Test and Microhardness Test

**Figure 2:**
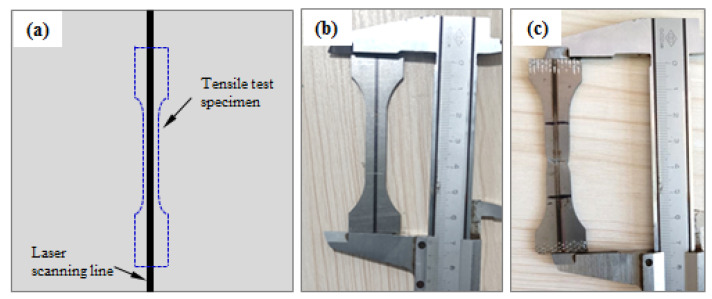
Cutting position and the photo of the tensile test specimen: (**a**) cutting position; (**b**) the photo of the specimen before the tensile test; (**c**) the photo of the specimen after the tensile test.

To investigate the effect of laser forming on the strength and plasticity of the specimens, monotonic tensile tests were performed at room temperature on a UTM-5105 universal testing system made by OBT company in Shenzhen, China. The testing items included yield strength, tensile strength, and elongation. The tensile specimens were cut using a DK7740HZ-B wire-cutting machine manufactured by Zhongyuan Machine company in Zhejiang, China, according to the standard of GB/T 228.1-2010: Metallic materials—Tensile testing—Part 1: Method of test at room temperature. Figure 2 shows the cutting position [21] and the photo of the tensile test specimen before and after the tensile test. The gauge length and width of the specimen were 30 and 8 mm, respectively. The extension rate applied in the tensile tests was 2 mm/min. Three repeated tests were performed for each combination of laser parameters. It is clear from Figure 2c that necking appeared, and the specimen failed at about the center of the gauge length.

**Figure 3:**
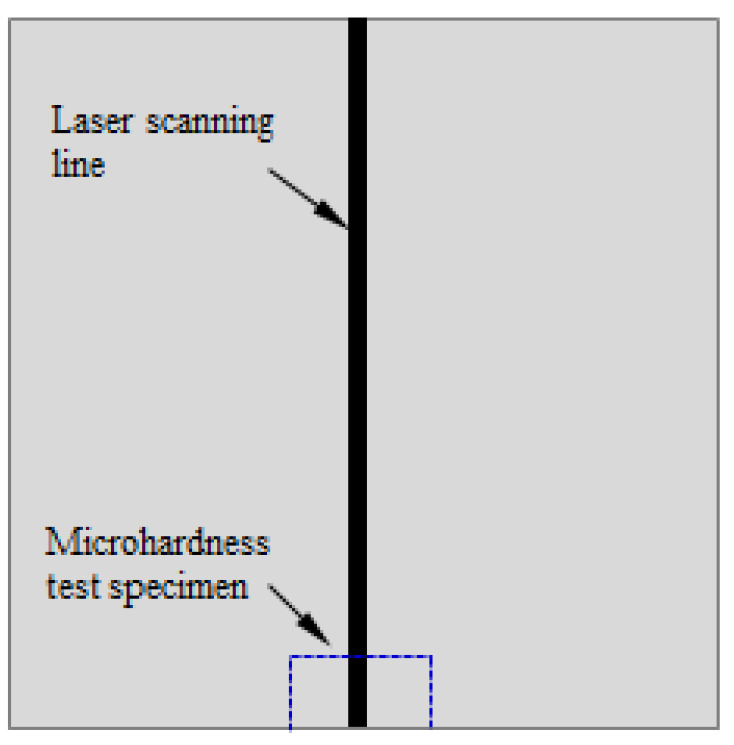
Schematic view of the specimen shape and cutting position for the microhardness and metallographic tests.

**Figure 4:**
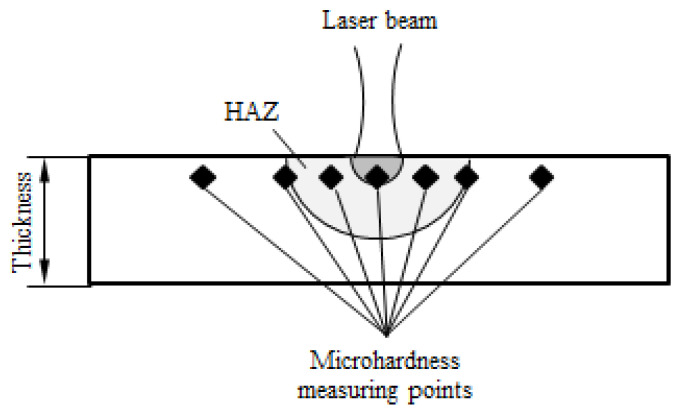
Schematic view of the cross-section and positions of the microhardness measuring points.

Vickers microhardness tests were conducted on an HV-1000 microhardness tester manufactured by FangYuan company in Haiyan, China to understand the hardness changes of the material before and after the laser-forming process. Rectangular specimens for microhardness and metallographic tests were cut from the laser-formed steel plates, and the cut sections were perpendicular to the laser-scanning lines, as shown in Figure 3. The cross-section of a laser-formed steel plate and the positions of the microhardness measuring points are shown in Figure 4. There was a total of seven measuring points, which covered the heat-affected zone (HAZ) and parent material of each specimen. The outermost two points were measured to determine the microhardness of the parent material, whereas the other five points (with intervals of 1 mm between each other) were measured to determine that of HAZ. A load of 0.2 kgf was applied for 15 s during the microhardness tests, and each point was measured three times to determine an average.

### 2.3. Metallographic Test

**Figure 5:**
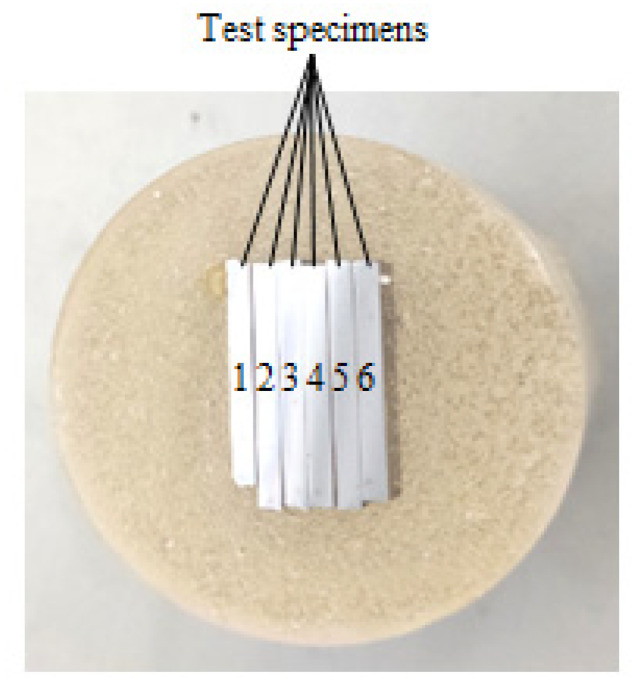
View of the specimens inlaid for the metallographic test.

To investigate the microstructural changes of the laser-formed DP980 steel plate, metallographic tests were performed using a 4XC-PC metallographic microscope made by ShangGuang company in Guangzhou, China. Due to their small size, every six specimens were inlaid together for convenience, as shown in Figure 5. After being completely polished, the specimens were etched in the ethanol solution containing nitric acid with a volume fraction of 4% for a few of seconds and then rinsed immediately and dried for microscopic observation.

## 3. Results and Discussion

### 3.1. Monotonic Tensile Properties

**Figure 6:**
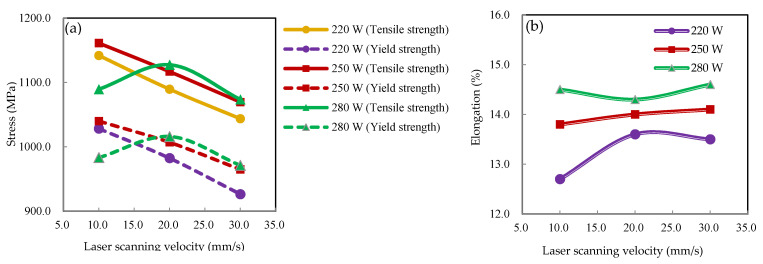
Monotonic tensile properties under different laser parameters: (**a**) yield strength and tensile strength; (**b**) elongation.

**Figure 7:**
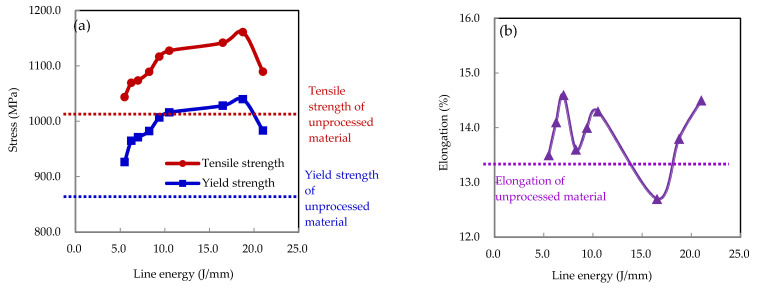
Monotonic tensile properties under different line energies: (**a**) yield strength and tensile strength; (**b**) elongation.

The monotonic tensile properties of the laser-formed specimens under different laser parameters as well as unprocessed ones were investigated through monotonic tensile tests, with the results shown in Table 2. To precisely evaluate the effects of laser power and scanning velocity on tensile properties, the yield strength, tensile strength, and elongation of the specimens were studied separately, as shown in Figure 6. It is noted from Figure 6a that the variation trends in the yield strength and tensile strength of the specimens under the same laser power conditions were almost the same. At laser powers of 220 and 250 W, both yield strength and tensile strength decreased with an increase in laser-scanning velocity. However, at a laser power of 280 W, the yield strength and tensile strength first increased and then decreased with an increase in laser-scanning velocity. In contrast, it can be observed that at the same laser-scanning velocity, both yield strength and tensile strength increased with an increase in laser power, except for combination 7 (280 W, 10 mm/s). This phenomenon may have been due to the line energy (21 J/mm) being near the melting line energy of DP980 steel [22], which may have induced a significant change in the microstructure of the material. Figure 7a shows the relationship between stress and line energy at different laser parameter combinations. It should be noted that after the laser-forming process, both the yield strength and tensile strength of the material were improved compared to the unprocessed one. Furthermore, both strengths increased with an increase in line energy before peaking at 18.75 J/mm and then dropping sharply. The yield strength and tensile strength maxima were 1040 and 1161.1 MPa—improvements of 20.6 and 15.1% compared to the unprocessed steel, respectively.

Elongations of the specimens processed under different laser parameter combinations are shown in Figure 6b. At a constant laser-scanning velocity, the elongation increased with an increase in laser power. However, no obvious changes occurred at a constant laser power, which indicated that the elongation of the material was insensitive to changes in laser-scanning velocity. Figure 7b shows the relationship between elongation and line energy, where the elongation of the material was improved slightly under almost all combinations of laser parameters. The largest value of elongation was 14.6%—an improvement of 9.0% compared to the unprocessed steel.

### 3.2. Microhardness

**Figure 8:**
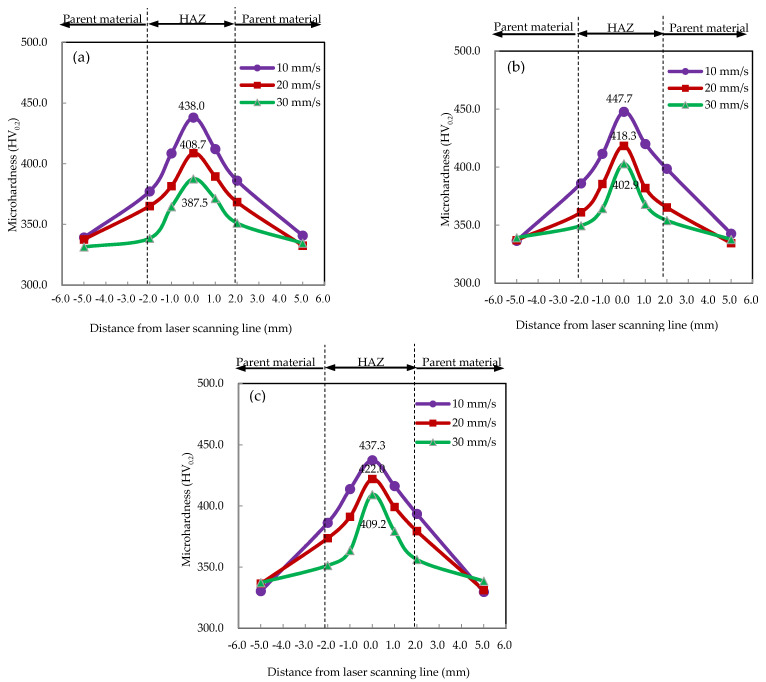
Microhardness distributions of the laser-formed plates under constant laser powers of (**a**) 220 W, (**b**) 250 W, and (**c**) 280 W.

Microhardness distributions of the HAZ and parent material under different laser parameter combinations are shown in Figure 8. According to Figure 8a–c, under a constant laser power, the microhardness of the same position decreased with an increase in laser-scanning velocity. Under the same laser parameter combination, the microhardness of the HAZ was much larger than that of the parent material—reaching a maximum at the laser-scanning line. When using the same laser-scanning velocity, as shown in Figure 8a–c, it was noted that the microhardness increased with an increase in laser power, except for combination 7 (280 W, 10 mm/s), which was consistent with the strength test results reported in Section 3.1. Furthermore, the microhardness difference between the cases of 10 mm/s and 30 mm/s decreased from 50.5 HV_0.2_ to 28.1 HV_0.2_ with an increase in laser power, respectively, which indicated that at a lower laser power, microhardness was sensitive to changes in laser-scanning velocity.

**Figure 9:**
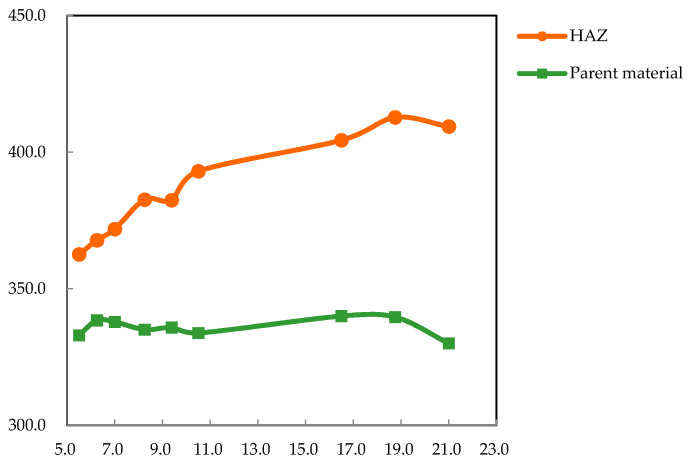
Relationship between the average microhardness and line energy for the HAZ and parent material.

Figure 9 shows the relationships between the average microhardness of the HAZ (as well as the parent material) and line energy. At a constant line energy, the average microhardness of the HAZ was much larger than that of the parent material. The average microhardness of the HAZ increased with an increase in line energy and reached a maximum of 412.8 ± 14.5 HV_0.2_—an improvement of 23.5% compared to the unprocessed material (334.2 ± 9.7 HV_0.2_). In contrast, the average microhardness of the parent material remained almost stable for different line energies and was close that of the unprocessed material.

### 3.3. Microstructure

**Figure 10:**
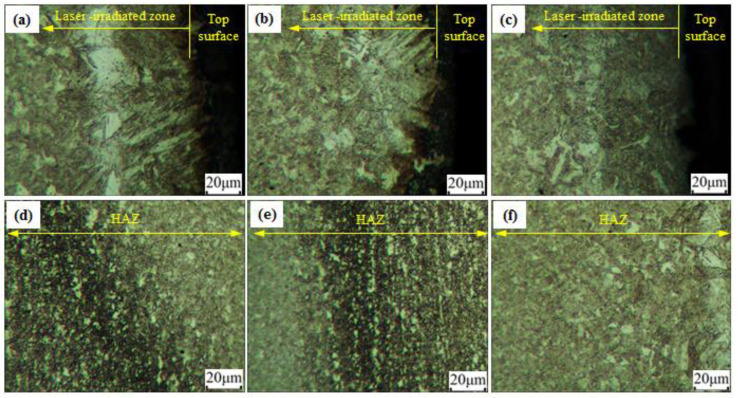
Microstructures in the thickness direction of the plate: (**a**) and (**d**) 280W, 10mm/s; (**b**) and (**e**) 280W, 20mm/s; (**c**) and (**f**) 280W, 30mm/s.

In order to explore the reason for the different strength behavior at 280 W, as shown in Figure 6a, the microstructures of the material formed under the different scanning velocity at 280 W were investigated, as shown in Figure 10. It was confirmed from Figure 10a–c that the grains in the laser-irradiated zone grew significantly at the laser-scanning velocity of 10 mm/s; there was little difference in the other two cases. However, in HAZ, as shown in Figure 10d–f, the grain size at the case of 30 mm/s was the largest. By contrast, the grain size in the case of 20 mm/s was smaller, which led to the higher strength.

**Figure 11:**
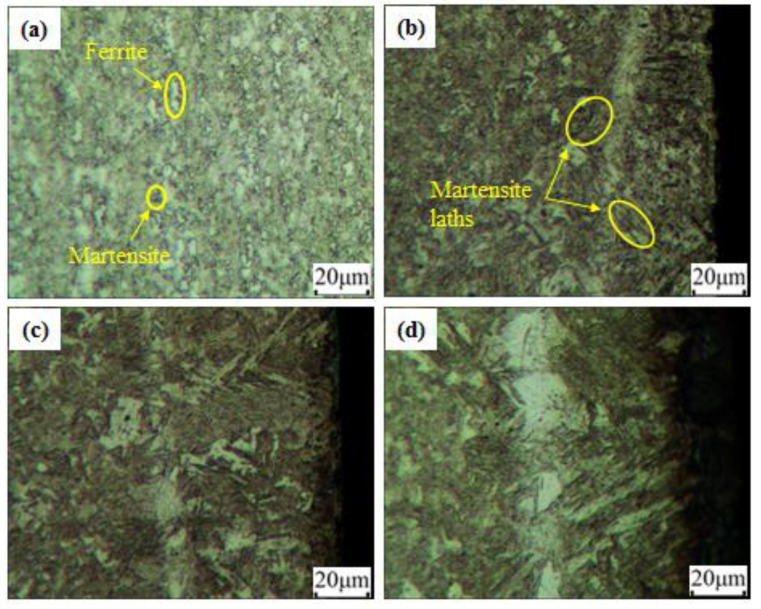
Microstructures of the laser-irradiated zone under different line energies: (**a**) 0 J/mm (unprocessed material); (**b**) 8.25 J/mm (combination 2); (**c**) 18.75 J/mm (combination 4); (**d**) 21 J/mm (combination 7).

Figure 11 shows the microstructures of the unprocessed material and the laser-irradiated zone of the laser-formed specimen. It was clear from Figure 11a that the microstructure of the unprocessed material consisted mainly of ferrite and martensite. Under a line energy of 8.25 J/mm (Figure 11b), thin and short martensite laths were observed, which enhanced the strength and microhardness of the material. When the line energy was increased to 18.75 J/mm (Figure 11c), it was found that the volume content of the martensite laths increased, which further enhanced the strength and microhardness. However, when the line energy was increased to 21 J/mm (Figure 11d), thick and long martensite laths were clearly observed and the grains grew rapidly due to the lower cooling rate, which is consistent with the research of Liu et al. [23]. The large grain size reduced the strength and microhardness of the material. Therefore, it was concluded that within a range of line energies below around 19 J/mm, the strength and microhardness of the DP980 steel increased with increasing line energy. Furthermore, the elongation of the material was also slightly improved by the laser-forming process.

## 4. Conclusions

The monotonic tensile properties and microstructures of DP980 high-strength steel processed under different laser parameter combinations were investigated experimentally. The following conclusions were obtained:

1. Both yield strength and tensile strength increased with increases in laser line energy below 19 J/mm due to the volume content increase of the martensite laths, and they then decreased due to the growth of the grains. The yield strength and tensile strength maxima were 1040 MPa and 1161.1 MPa, which corresponded to improvements of 20.6% and 15.1% compared to the unprocessed steel, respectively.
2. The elongation of the laser-formed material was slightly improved due to the existence of residual austenite, but no clear trend was observed between the elongation and laser line energy.
3. The average microhardness of the HAZ of the laser-formed material increased with increasing laser line energy and peaked at 412.8 HV_0.2_—an improvement of 23.5% compared to the unprocessed material.

## Figures and Tables

**Table 1:** Combinations of laser parameters for the experiment.

Combination No.	Laser Power (W)	Scanning Velocity (mm/s)	Line Energy (J/mm)
1	220	10	16.50
2	220	20	8.25
3	220	30	5.50
4	250	10	18.75
5	250	20	9.38
6	250	30	6.25
7	280	10	21.00
8	280	20	10.50
9	280	30	7.00

**Table 2:** Monotonic tensile properties of the specimens.

Combination No.	Yield Strength (MPa)	Tensile Strength (MPa)	Elongation (%)
1	1028.3 ± 26.1	1141.9 ± 26.4	12.7 ± 0.4
2	982.4 ± 24.6	1089.5 ± 27.5	13.6 ± 0.3
3	926.4 ± 17.7	1043.7 ± 20.5	13.5 ± 0.4
4	1040 ± 25.2	1161.1 ± 25.4	13.8 ± 0.4
5	1007 ± 26.9	1116.8 ± 30.7	14.0 ± 0.3
6	964.9 ± 27.3	1069.5 ± 30.8	14.1 ± 0.3
7	983.3 ± 25.8	1089.6 ± 24.1	14.5 ± 0.3
8	1016.1 ± 28.8	1127.5 ± 31.1	14.3 ± 0.5
9	971.2 ± 22.4	1073.7 ± 21.5	14.6 ± 0.2
Unprocessed material	862.3 ± 15.7	1009.2±17.4	13.4 ± 0.3

## Data Availability

Not applicable.
